# Some Dental History

**Published:** 1902-10-15

**Authors:** A. T. Metcalf

**Affiliations:** Battle Creek, Mich.


DENTAL REGISTER.

Vol. LVI. October 15, 1902. No. 10.

### SOME DENTAL HISTORY.

BY DR. A. T. METCALF, BATTLE CREEK, MICH.

Being an Address delivered to the Graduates of the Dental Department of the University of Michigan.

When I accepted the kindly invitation to address you on this occasion, I realized the fact that I had several times been honored with a similar privilege by former classes, so I requested your president to name some topic that might be of special interest to you at this time. As he suggested that something historical would be acceptable, and as this is a subject that is always of interest to me, I hope that, for the little time I shall occupy your time, you will not be entirely unconcerned.

If I should undertake to address you upon the dentistry of to-day, I would have to take at least one course at the college in order to do so intelligently. Having been out of practice for a number of years, and the profession having advanced in knowledge and material equipment, I confess to being a back number. I was more deeply and profoundly impressed with my ignorance when I picked up a circular the other day in a dental office, containing instructions in relation to mumification of nerves, and advising "Pulp Conservator and Anæcologen" as remedies that never fail to produce satisfactory results. I do not suppose that all of you have seen this circular. While I am conscious of neglecting a duty in not reading it to you, I will take the time to read the postscript which is of such a kindly nature it would be unfair to withhold it:

"P. S.—It is my wish to make as large a return to you as I can for your money and courtesy. Therefore, please study over the directions sent with Anæcologen, once a week, for three months, as one forgets so easily, and there is such an immense curative field covered by Anæcologen. Please remember these suggestions."

Mumification of *dead* bodies usually shows a great amount of shrinkage of the tissues, and it is hardly probable that live tissue can be mumified without contraction. If the latter premise is correct, what condition will the nerve channel be left in after the nerve is mumified? But this circular states that the nerve, by mumification, has been turned into leather, or a leathery substance. As these results are a *sure thing*, according to the circular, it must have been a great sacrifice to have extracted a good tooth—one that had been saved by mumification of the nerve—to ascertain that leathery condition. I do not pretend to deny anything the circular sets forth, and only mention it to show my ignorance, and how I have fallen behind in the fourteen years I have been out of practice. After a careful perusal of the circular, I confess to a peculiar sensation creeping over me, but whether paralysis or a partial mumification, I have been unable to determine. When I was in practice, a dead nerve was considered as dead as any other tissue, and quite liable to decomposition.

How fortunate it is for us that it can be mumified and turned into leather!

I realize that you are about to leave these walls where you have spent many months in preparation for the practice of one of the most useful and honorable professions; that you will have earned the laurels conferred by your diplomas; that you are anxious to go to your homes, and that speeches and addresses at this time can not excite more than a half interest. You have been satiated with lectures, instruction and advice, until you wish I had not come. But I am here to perform a pleasing duty (pleasing to me), and if it is irksome to you, bear in mind that the end is near and that you will soon be free.

Dentistry as a generally acknowledged profession has had an existence of less than three-fourths of a century. While it is true that there were treatises on the teeth, their value and their beauty centuries ago, the subject was treated anatomically and theoretically rather than practically. Their importance in giving expression to the human face is referred to several times in the Bible. Jacob, in blessing Judah, says: "His teeth shall be white with milk;" and in the Songs of Solomon we find that he compares a fine set of teeth to a "flock of sheep even shorn that come up out of the washing, every one bearing twins."

It has been stated that mineral artificial teeth were manufactured by a Frenchman as early as 1776; but the fact that a Frenchman came to this country at a subsequent period and manufactured a full upper and lower set of teeth, made from natural teeth strung together by gold wires, disproves the statement. This set of teeth was made for General Washington, and I had them in my possession several years. They were destroyed in a fire at New York City in 1854.

It was not until 1835 that mineral artificial teeth were manufactured for the supply of dentists, and then by only one manufacturer. To-day there are a number of establishments engaged in their manufacture, and are producing results, that, in the hands of skillful dentists, can be so arranged as to replace the natural organs, defying detection.

While it has taken many years to bring about these wonderful results in supplying artificial dentures, operative dentistry has advanced to still greater and more marvelous attainments, and which could have been brought about in no other way than by associated effort. I can not undertake in this brief paper to show that no other department of human activity and usefulness has made greater progress in the same number of years. I shall simply try to point out a few facts to show that our State of Michigan has been at the forefront, and performed her full share in the general improvement and advancement. The first and perhaps the most important move in the right direction was the organization of our State Dental Society, in January, 1855. There were present thirteen dentists (so called), not one of whom was a graduate of a dental college. With possibly two exceptions, including myself, these pioneers in dentistry in this State have gone down into the shadows.

At a meeting of this society in 1881, a committee was appointed to collect material and write the history of dentistry in Michigan, and present it at the next meeting of the society, which was held at the Russell House in the city of Detroit. This meeting was held on the 31st day of March, 1882, was largely attended, and was the most important and interesting meeting of the society up to that time. There were present a number of distinguished dentists from abroad, including Dr. Taft, then of Cincinnati; Drs. Barrett, of Buffalo, Brophy, Talbot, Harlan, and Allport, of Chicago, and others. Among the letters of regret I want to read you one—a characteristic letter from a great, good man and dentist, and I believe at one time partner of Prof. Taft. He was not well enough to attend:

"XENIA, O., March 27th, 1882.

"*Dear Friends:*—The milder weather has done something, but not enough. I can't go. From neck down to toes there is nearly constant toothache, or something equivalent; my head is easy enough to go, but it is fastened on, so I can't send it. If you find a heart, some affection, and several strong inclinations running about loose, count them mine. Indeed, if you find a stray spirit of a rather friendly cast lingering near, like Mary's little lamb, treat it kindly for my sake. My dilapidated body will not be with you.

"May our Father in Heaven bless you all.

"Your brother,

"GEO. WATT."

I have read this letter not only as a curiosity in its way, but for the purpose of showing the brotherly spirit that universally prevailed among the members of the profession at that time—a general spirit of interest and helpfulness.

The committee appointed at the previous annual meeting read a lengthy, complete and very interesting history of dentistry in this State, which was afterward printed in full in the Transactions of the Michigan Dental Society of that year. In no other way can the progress of the profession be shown so well as a completion of the history to the present time, and it is a matter that ought to receive attention.

The time was—and within my recollection—when every dentist was a "law unto himself;" when a new improvement in manipulation was kept as a secret and obtained only by purchase. To-day, by means of our associations and colleges, everything in the line of improvement in manipulation or material is poured into the common flood of knowledge, so that very few claim superiority. Those who do claim distinction are a cheap lot—a fraud and a disgrace to the profession and to humanity.

As all professions are disgraced with imposters, the dental profession does not stand alone. You need not be discouraged because of them, for by their incompetence they make business for you. It is a safe prediction, and one that never fails, that anyone, claiming to be a professional man, who advertises himself as superior to others, or having superior advantages, and is not only willing but anxious to confer these advantages upon an unsuspecting public at less cost than a good article can be obtained elsewhere, is not only an incompetent, but of the same species that invites the innocent fly into its parlor.

You very naturally inquire, what we did to alleviate suffering in extracting teeth and other surgical operations before chloroform and ether came to our assistance. I do not know what others may have done, but in my office we practiced what is now called hypnotism, psychology, mesmerism, animal magnetism, Christian science, mental healing and all that sort of thing.

The comparatively new fads, mind cure, faith cure, etc., however well they work in some hands and in some cases, don't appear to be a very great success in the hands of the dentist. When the dentist begins to tickle that little fiber of the tooth called the nerve, somehow or other it is impossible to make people believe that it don't hurt—that it is all in their minds; and it has been my experience that those who preach and practice these delusions are the most fidgety and troublesome patients I ever had. I do not mean to be understood as saying that there is no virtue in mental healing or mind cure, or Christian science, or whatever you may choose to call this practice, for we all know that there are many cases of illness, or supposed illness—mental aberrations, perhaps—that can be more successfully treated by this means than by drugs.

When I commenced practice, filling teeth with gold was something of a secret, known to comparatively few. I well remember that the first really beautiful fillings that I ever saw were made by a Dr. Lord, of Georgia. He was in the habit of spending a month or two every summer in a little country town ten or twelve miles from the city of Utica, where I resided. He would not give away his method of filling with gold, and my instructor was so anxious to come into its possession, that we concocted a successful scheme to filch it from him without his knowing it, We realized that we had accomplished a good thing, but I shall not endanger my reputation by relating how we secured the prize.

About the time of which I have been speaking—the time when we used no other than soft foil—sponge gold made its appearance, and was made in the office of Dr. H. R. White, in Utica, N. Y. The first article on the subject of sponge gold emanated from his office, and was published in one of the dental journals of that day. I have mentioned this fact for two reasons, first, because Dr. White, the inventor, came to this State several years afterwards, practiced a little in this city, and died here; and secondly, because it is generally supposed that A. J. Watts was the inventor. He invented no part of the manufacture of sponge gold, but he did secure a patent for it while he was employed by Dr. White in its manufacture. Dr. White is dead. If there is any honor or credit to be given for the invention of this form of gold filling, let it rest where it belongs. I stand here ready to prove, without leaving a shadow of a doubt, who was the inventor, and who prepared the first paper that appeared in any dental journal in the country on the subject. With the advent of sponge gold came the necessity for serrated instruments. Up to this time our pluggers were sharp-pointed or smooth, and many of our best operators used a single-pointed instrument. Think of it—of the time required to put in a large filling with instruments having only a single point! Such were the instruments of some of our most noted dentists of that time. As the sponge gold required serrated instruments to manipulate it successfully, pluggers were made out of hand burrs of different sizes, and bent at different angles.

Since the dental engine has become an instrument of universal use, I presume the dentists of to-day know nothing whatever of the old-fashioned hand burrs that we rotated between the thumb and fingers, requiring minutes to do what the engine will do easily and without any note of time whatever. To make these hand burrs into pluggers the wire of the handle must be enlarged in some way so that they could be held firmly in the hand. All sorts of devices were resorted to for this purpose, but I will mention only one—that used by me, but not my invention. I would take pieces of sole leather of the proper size, punch a small hole through the center of each one, then drive the wire handle of the burr through each piece until I had pieces enough on the wire handle of the burr for the desired length ; then with a sharp knife trim and dress the leather to suit the hand. Soon serrated instruments, suitable for the purpose, were placed on the market, but they were clumsy affairs as compared with the symmetrical, perfectly serrated and beautifully finished instruments of to-day. I have dug out of the debris of many years a couple of home-made serrated pluggers, which are a curiosity to-day, and I will leave them for the museum, provided the faculty will assure me they shall be securely placed where they can not do any damage.

The next step forward was the annealing or heating of soft foil, and by that means imparting to it an adhesive quality—the heating was supposed to throw out spicula on the surface, so that by pressing separate pieces or pellets together they would become so interlocked or united, that separation became impossible. For the purpose of annealing, an annealing lamp, a compound blow-pipe, was placed on the market, giving any desired heat up to the melting point, which met with a ready sale. Such was the demand for the lamp that the manufacturer could not make them fast enough to meet it. The discovery of making gold adhesive by annealing was made by a dentist of this State, and by the individual who placed on the market the first annealing lamp.

But, with the improvements made in dentistry for the last fifty years, none met with greater favor than the dental engine. As a labor-saving device, I believe it is looked upon as the *ne-plus* of all inventions for dental purposes. The first dental engine was a crude affair, but for three or four years it answered an excellent purpose, and met with a ready sale. It was a pneumatic—

I came very near saying rheumatic—engine, the power created by a double-acting bellows worked by the foot. Besides a rotary motion, by means of a cam, it had a lateral, or to and fro motion, used as a file-carrier for separating the teeth and finishing approximal fillings. It had also a right-angle attachment. This engine, the first one made, has been placed in the dental museum. (This engine was made by a man by the name of Green, of Kalamazoo, Mich., under the direction of Dr. Metcalf.—Ed.)

So far as I can call to mind, the first pneumatic plugger placed on the market was a Michigan product, patented in 1866, and was publicly exhibited for the first time at a meeting of the State association held in Adrian in 1867.

Besides the inventions already mentioned as the products of Michigan dentists, we claim text-books by Dr. Taft, instruments by Dr. Watling, appliances by Dr. Dorrance, discs and mandrels by Dr. Moore, and others that ought to be mentioned in this connection.

This dental college is the outgrowth of the work and influence of our State association. That much maligned but very useful body, the State Board of Examiners in Dentistry, is also the outgrowth of our State Dental Association. It is a powerful adjunct to our colleges and a menace to those who engage in practice without the proper credentials. When that board was organized there were a large number of incompetents engaged in practice in this State. To-day there are comparatively few.

I have now occupied all the time allotted to me, but have said very little on the subject I started out with. You may think it a good deal like the lecture of Artemus Ward entitled "Sixty Minutes in Africa." In this lecture of sixty minutes, the only allusion he made to Africa was this: "I have never been there, but I understand that the hotels of Africa are conducted on the African plan."

What I have said about improvements and advancement in dentistry comprise the best part ot the history of dentistry in this State. I have said enough to convince you that the dentists of Michigan are entitled to no small share in its development and the position our profession occupies among other professions of to-day.

And now just a few plain words in conclusion. Not that I expect to say anything new to you, but to strongly impress upon your minds certain things, which, if scrupulously observed, will insure you your full share of patronage—presuming, of course, that you are fully qualified for the practice of your profession. There are two prerequisites to a good practice. They are simple, inexpensive; in fact cost nothing beyond a little cultivation. They are courtesy and affability—not that mawkish courtesy that mocks gentility, nor that affability which breeds contempt—but that courtesy and affability that marks the perfect gentleman.

There is one special duty that must never be neglected and that is cleanliness—cleanliness of both body and mind. It is not only necessary that you should wash your hands before touching your patient, but it is necessary that you should lave them in the presence of your patients, that they may have ocular assurance that your hands are clean. If I was successful in my practice, I believe it was due more to my well known scrupulous care in this direction than from any supposed ability I may have possessed.

You must not presume that because you go hence with a certificate of graduation that people will flock to you in great numbers to secure your valuable services. I hope none of you will be disappointed. If you are, do not let the world know it; but keep at work, be diligent, remembering that if you would reap to-morrow you must sow to-day. He who plows and harrows and sows and tends and cares, need not worry as to the harvest. It will come in due time. Do not be led astray by any alluring hopes of prosperity by advertising yourselves superior to your fellows, as the possessors of wonderful remedies or appliances not known to others. Be honest with yourselves, true to your profession, and loyal to your Alma Mater, and you will earn a good name and wear a crown of success. But these can not be obtained without work. So my last words to you are, Work! Work!!

